# Does Postoperative Mechanical Axis Alignment Have an Effect on Clinical Outcome of Primary Total Knee Arthroplasty? A Retrospective Cohort Study

**DOI:** 10.2174/1874325001711011330

**Published:** 2017-11-29

**Authors:** Mikhail Salzmann, Peter Fennema, Roland Becker, Hagen Hommel

**Affiliations:** 1 Hochschulklinikum der MHB Theodor Fontane, Städtisches Klinikum Brandenburg GmbH, Center of Orthopedics and Traumatology, Hochstraße 29, 14770 Brandenburg an der Havel, Germany; 2 AMR Advanced Medical Research GmbH, Hofenstrasse 89b, 8708 Männedorf, Switzerland; 3 KH-MOL GmBH Sonnenburger Weg 3, 16269 Wriezen, Germany; 4 Medizinische Hochschule Brandenburg (MHB), Theodor Fontane, Fehrbelliner Straße 38, 16816 Neuruppin, Germany

**Keywords:** Osteoarthritis, knee, Total knee arthroplasty, Constitutional varus, Clinical outcome, Surgical accuracy, Retrospective cohort study

## Abstract

**Background:**

There is an ongoing debate whether patients with constitutional varus should be restored to neutral mechanical alignment following total knee arthroplasty (TKA).

**Objective:**

The aim of this retrospective cohort study is to determine whether mild unintentional postoperative varus alignment (3°–6°) influences TKA outcome in patients with and without preoperative varus alignment due to medial osteoarthritis of the knee.

**Methods:**

We analyzed 172 consecutive TKA cases between April 2011 and May 2014. Patients were divided into four groups based on their preoperative and postoperative hip-knee-ankle angles (HKA): preoperative varus ≤ 3° with postoperative varus position ≤ 3° (Group 1, n = 47); preoperative varus >3° with postoperative varus ≤ 3° (Group 2, n = 104); preoperative varus ≤ 3° with postoperative varus malalignment > 3° (Group 3, n = 3); and preoperative varus > 3° with postoperative varus malalignment > 3° (Group 4, n = 18). Patients were followed up until 2 years postoperatively.

**Results:**

Knee Society Score and Western Ontario and McMaster University Osteoarthritis Index scores for all study groups increased following TKA, with no postoperative differences at any time point. Group 4 performed significantly better on the Forgotten Joint Score than Group 2 (p = 0.019). Group 4 performed significantly better on the High Flexion Knee Score than Group 2 (p = 0.004) and Group 1 (p = 0.019). All other between-group differences were not statistically significant.

**Conclusion:**

Residual postoperative varus alignment of the lower limb does not appear to adversely affect clinical outcome following TKA for varus-type osteoarthritis.

## 1 INTRODUCTION

During the last few decades, there has been consensus that restoration of neutral limb alignment is necessary for successful total knee arthroplasty (TKA) outcome. Previous studies have found that inadequate restoration of leg alignment has an adverse effect on implant survivorship [1-4]. A hip-knee-ankle angle (HKA) of 0° ± 3° is generally considered necessary to avoid implant failure in the medium- or long-term [5, 6].

Recent research has shown that a natural varus limb alignment is present in a relevant proportion of the physiologically normal human population. A study by Bellemans *et al.* showed that this native varus alignment is present in approximately 17% of women and 32% of men, which they defined as constitutional varus [7]. For patients with constitutional varus who require TKA later in life, restoration of their constitutional alignment could be a better option than restoration to a neutral mechanical alignment. Matziolis *et al.*, who conducted a matched cohort study to compare patients with unintentional residual varus and patients with neutral mechanical alignment, found a Knee Society Score (KSS) of 158 points in the varus group and 142 points in neutral alignment group (p > 0.05). No significant differences were found on the Western Ontario and McMaster Universities Arthritis Index (WOMAC) and the Short-Form 36. Another study by Vanlommel *et al.* found that undercorrection of varus deformity improved function [8]. The researchers found that TKAs placed in mild varus scored significantly better at 7 years on the KSS (with 10 points difference on the KSS) and WOMAC (with 15 points difference), as compared with knees that were corrected to neutral alignment [8].

A more recent study published by Meneghini *et al.* contradicts these findings: upon final follow-up at 1 year, higher KSS - Knee Scores (KSS-KS) were found in varus knees corrected to neutral alignment than in varus knees that were left in varus position or corrected to valgus (p = 0.025) [9]. However, these differences were marginally small (with KSS-KS of 97, 95 and 93, respectively), and *post hoc* comparisons were not statistically significant.

Since the effect of residual malalignment on clinical and functional outcome is contradicting, and there is conjecture whether postoperative varus alignment yields clinical advantages, there is an ongoing need for further research in the field [10]. We, therefore, designed and conducted a study to determine whether neutral postoperative alignment yields better early postoperative clinical outcome, as compared with mild unintentional postoperative varus alignment (3°–6°) following TKA. To determine whether this association would be modified by preoperative alignment, we assessed the association in patients with and without preoperative varus malalignment.

## 2 MATERIALS AND METHODS

Between April 2011 and May 2014, 248 patients received a Journey II BCS posterior stabilized prosthesis (Smith & Nephew Inc., Memphis, TN). All surgical procedures were performed by one surgical team, consisting of four senior orthopedic surgeons, at a single institution.

The study cohort comprised patients with medial primary gonarthrosis and varus alignment as the underlying indication for TKA. After applying the study's exclusion criteria, the study population consisted of 172 consecutive patients (172 TKAs; Table **1**). Excluded for the study were patients with preoperative valgus alignment of the leg (n = 19), clinical scores not available (n = 18), patient refusal to provide informed consent (n = 19), patient death for reasons unrelated to the TKA procedure (n = 6), and lost to follow-up (n = 14). Patients who underwent a device explantation were included until the time point of reoperation.

Standard instrumentation was used for the proximal tibial and the distal femoral cuts. The “extension gap-first technique” was used to perform gap balancing with a balancer device [11, 12]. A balancer device was used to distract the femur from the proximal tibia. Following each soft-tissue release step, the device was used to measure the extension gap until a rectangular extension gap was obtained. A gradual soft-tissue release was carried out to obtain a symmetrical extension gap if required [13, 14]. The values established for the extension gap were then applied to the flexion gap. A rectangular flexion gap was achieved by femoral rotation, which was based on the tension of the soft-tissues. All participating surgeons employed the same surgical technique.

The HKA was measured preoperatively and postoperatively by a physician who was blinded to the patient’s clinical information and prior radiology and who was unaware of the study. HKA angles were obtained on full length, weight-bearing radiographs with subjects standing barefoot and the patellae oriented forward [15]. For mechanical alignment, a deviation of ±3° from neutral alignment was considered the normal range [5, 6]. Therefore, malalignment was defined as varus mechanical alignment of less than 177° and valgus mechanical alignment of more than 183°. HKA angles were measured from digital radiographs using a dedicated measurement tool of the software package mediCAD (mediCAD Hectec, Altdorf, Germany).

The radiographs and the clinical scores, including the KSS-KS and the KSS – Function Score (KSS-FS) [16] and the Western Ontario and McMaster University Osteoarthritis Index (WOMAC) [17], were prospectively determined preoperatively and postoperatively at 1 and 2 years postoperatively, and through the Forgotten Joint Score (FJS) [18] and the High Flexion Knee Score (HFKS) [19] at 2 years.

Patients were divided into four groups based on preoperative and postoperative HKA: Group 1 had a preoperative varus ≤ 3° and postoperative varus position ≤ 3° (n = 47); Group 2 had a preoperative varus >3° and postoperative varus ≤ 3° (n = 104); Group 3 had preoperative varus ≤ 3° and postoperative varus malalignment > 3° (n = 3). Group 4 had preoperative varus position > 3° and postoperative varus malalignment > 3° (n = 18). The prevalence of preoperative and postoperative mild varus malalignment in our study population was 70.9% and 12.2%, respectively. None of the patients had a postoperative malalignment of more than 6°.

Statistical analysis was performed using the Stata 12.1 (StataCorp, College Station, TX). Patient demographic data, clinical scores, and preoperative and postoperative radiographic leg alignment data were registered as the mean and the standard deviation. A one-way ANOVA test was used to compare the continuous variables amongst the 4 groups, using the Bonferroni multiple-comparison test for the post-hoc pairwise comparisons. Categorical variables were compared employing the Fisher’s exact test. Implant survivorship was calculated using Kaplan-Meier analysis [20], with the following events of interest: implant revision due to any reason, and implant revision due to aseptic loosening. Logrank tests were used to determine the presence of between-group differences in terms of implant survival. P-values less than 0.05 were considered significant.

## 3 RESULTS

There were 50 patients with a preoperative neutral alignment, with an average alignment of 1.9° ± 1.0°. Of those, 47 patients were corrected from 1.9° ± 1.1° preoperative to 0.8° ± 1.0° postoperative, and 3 patients were corrected from 2.3° ± 0.6° preoperative to 4.7° ± 0.3° postoperative (Table **2**).

There were 122 patients with a preoperative varus alignment, with an average alignment of 7.4° ± 3.4°. Of those, 104 patients were corrected from 7.0° ± 2.3° preoperative to 1.4° ± 1.0° postoperative, and 18 patients were corrected from 10.1° ± 3.4° preoperative to 4.4° ± 0.8° postoperative.

During the course of the study, 5 patients with a preoperative varus and a postoperative neutral alignment were revised for aseptic (n = 4) and septic (n = 1) loosening. Mean time to revision was 17.4 ± 3.7 months (range, 12 – 22 months). In addition, one aseptic loosening of the tibial baseplate was found in a patient with a preoperative varus of 13° and a postoperative varus of 5° at 2 years. Implant survival rates are presented in Table **2**. Postoperative complications were one hematoma, one suspected infection, one wound healing disturbance in the preoperative varus alignment group, whereas no complications were noted in the cohort with a neutral preoperative alignment. In the cohort of patients with preoperative varus and postoperative malalignment, a non-progressive radiolucent line was noted under the tibial component in one of the 18 knees (5.6%).

Preoperative scores did not differ significantly between the four groups (Table **2**). KSS and WOMAC scores increased following TKA for all study groups, with no postoperative differences at any time point (Table **2**). The patient group with preoperative varus and postoperative malalignment (Group 4) scored significantly better in the FJS as compared with the preoperative varus and postoperative neutral group (Group 2) (p = 0.019); the other between-group differences were not statistically significant. The patient group with preoperative varus and postoperative malalignment (Group 4) scored also significantly better in the HFKS when compared with the preoperative varus and postoperative neutral group (Group 2) (p = 0.004) and with the preoperative neutral and postoperative neutral group (Group 1) (p = 0.019); the other between-group differences were not statistically significant.

## 4 DISCUSSION

The primary finding of this study is that both postoperative mild varus and neutral mechanical alignment of the lower limb can lead to excellent functional outcomes. With a surgical goal of neutral mechanical alignment, there appears to be no difference in postoperative functional outcome between patients with and without preoperative mild varus malalignment. The current study suggests that residual postoperative varus alignment of the lower limb is acceptable following TKA for varus-type osteoarthritis. Unintentional undercorrection of the varus deformity during TKA was associated with better FJS and HFKS, but not with better KSS and WOMAC scores.

There is consensus that restoration of neutral limb alignment is a prerequisite for successful TKA [21]. Limb alignment is an important variable that is determined by the surgeon and impacts postoperative functional and survival outcomes. The current gold standard in the implantation of a TKA is an HKA of 180° ± 3° [10]. The alignment of the components outside this safe zone may be associated with a poor clinical outcome [14] and lower implant survival. Ritter *et al.* investigated 6,070 TKAs with a mean follow-up of 8 years and found an increased revision rate for knee joints with postoperative varus alignment (tibiofemoral axis, <2.5°) and valgus positions (tibiofemoral axis, >7.5°) [4, 22]. Similar results were reported by Kim *et al.*, who examined 3,048 TKAs with a mean follow-up of 16 years [23]. The authors found a revision rate of 2.3% for postoperative varus malalignment (tibiofemoral axis, <3°) as compared to a 0.6% revision rate in the neutrally aligned knees (tibiofemoral axis, 3°-7.5°). There was no significant increase in the revision rates for valgus deficiencies (0.9%) [23]. An important finding from the latter study, which was not assessed in the present study, was that correct alignment requires both a neutral orientation of the femur as well as the tibial component [22]. The compensation of a varus or valgus orientation of the one component by the other leads to a significantly increased failure rate of 3.2% to 7.8% [22].

However, more current studies found no correlation between positioning and revision rate. Bonner *et al.*’s study of 501 TKAs with 15-year follow-up [24] and Parratte *et al.*’s study of 398 patients with 15-year follow-up [25] did not find any increased revision rates for prostheses with a postoperative orientation of the mechanical axis greater than 3° varus. An analysis of the literature shows that more recent papers (published since 2010) indicate that residual varus alignment of the lower limb do not necessarily lead to an increased failure rate of the implant [8, 25-27], with incidental studies showing undercorrection of a varus deformity yielding clinical advantages [8]. A possible explanation for these findings may be that overall mean alignment of the Caucasian population might be slightly varus. For this reason, undercorrection would align these patients to their pre-disease status [7, 28]. A major drawback of this concept is that the pre-disease alignment for the individual patient is typically unknown. Varus alignment determined in the osteoarthritic patient prior to TKA is the resultant of pre-disease alignment alongside a year-long process of degeneration of bone and articular cartilage. Only longitudinal studies starting in the pre-disease stage will enable us to determine the relationship between pre-disease and pre- and post-arthroplasty alignment.

The present study was not designed to assess the impact of unintentional varus alignment on implant longevity, which is a major shortcoming. Another limitation was that the individual femoral and tibial component alignment was not measured. Several studies have established an association between component alignment and clinical outcome [26, 29, 30]. Another shortcoming was that we were unable to determine whether any differences exist between mild and severe varus, as previously suggested by Vanlommel *et al.* [8]. Next, as already stated by Vanlommel *et al.*, the outcome variable KSS-KS is linked with the exposure variable, as the KSS-KS awards more points to knees with neutral alignment, which lowered the KSS-KS in the mild varus group [8]. Other limitations were the small number of cases, especially in the group with preoperative neutral alignment and postoperative mild malalignment, the relatively large proportion of patient lost to follow-up, and the short follow-up time. The proportion of patients with postoperative residual varus alignment was small, which affects the probability that the significant findings reflect a true effect. We cannot preclude that the significant advantages in terms of postoperative FJS and HFKS may be the result of chance, or, due to the observational nature of the study, to confounding or bias. For this reason, a causal interpretation of the found associations is preliminary. A further limitation is that discretization of mechanical alignment may have introduced bias or loss of study power [31]. Finally, a single, prosthetic-guided motion knee design was used in all cases, and all knees where operated on through an extension-first technique. It is unknown whether our findings are generalizable to other knee designs and other surgical techniques. However, a conservative interpretation of our study findings is that postoperative mild varus does not appear to be associated with adverse functional outcomes, which is consistent with the findings of previous research [8, 27, 32]. However, due to the aforementioned limitations, as well as the limitations of previous studies, there is currently insufficient evidence to recommend a change of clinical practice. We, therefore, recommend that intentional undercorrection to 3° to 6° of varus only takes place within the framework of well-designed clinical studies.

## CONCLUSION

A conservative interpretation of our study findings is that postoperative mild varus does not appear to be associated with adverse functional outcomes, which is consistent with the findings of previous research [8, 27, 32]. However, due to the aforementioned limitations, as well as the limitations of previous studies, there is currently insufficient evidence to recommend a change of clinical practice. We, therefore, recommend that intentional undercorrection to 3° to 6° of varus only takes place within the framework of well-designed clinical studies.

## Figures and Tables

**Table 1:** Baseline characteristics of the study cohort.

–	Group 1 Preop Neutral Postop Neutral (n = 47)	Group 2 Preop Varus Postop Neutral (n = 104)	Group 3 Preop Neutral Postop Malaligned (n = 3)	Group 4 Preop Varus Postop Malaligned (n = 18)	p-Value
Age [Years]	68.8 ± 6.3	69.5 ± 6.3	72.5 ± 3.0	69.6 ± 5.9	0.755
Sex (Female)*	27 (57.5)	58 (55.8)	2 (66.7)	11 (61.1)	0.966
BMI	30.0 ± 3.7	29.8 ± 3.9	30.0 ± 1.0	29.7 ± 4.3	0.979
ASA	1.6 ± 0.6	1.7 ± 0.6	1.7 ± 0.6	1.9 ± 0.5	0.398

Presented as mean ± standard deviation (range), except * presented as n (%). Abbreviations: preop, preoperative; postop, postoperative

**Table 2:** Study outcomes.

–	–	Group 1 Preop Neutral postop Neutral (n = 47)	Group 2 Preop Varus Postop Neutral (n = 104)	Group 3 Preop Neutral Postop Malaligned (n = 3)	Group 4 Preop Varus Postop Malaligned (n = 18)	p-Value
HKA	Preoperative	1.8 ± 1.0 (0.0 – 3.0)	7.0 ± 2.3 (4.0 – 16.0)	2.3 ± 0.6 (2.0 – 3.0)	10.1 ± 3.4 (4.0 – 15.0)	n.r.
	Postoperative	0.8 ± 1.0 (-2.5 – 2.0)	1.4 ± 1.0 (-2.0 – 3.0)	4.7 ± 0.3 (4.5 – 5.0)	4.3 ± 0.8 (3.5 – 5.5)	n.r.
KSS-KS	Preoperative	24.3 ± 5.0	25.2 ± 3.7	28.7 ± 3.8	26.1 ± 5.0	0.186
	1 year	89.0 ± 3.4	89.4 ± 4.4	86.7 ± 9.2	88.8± 5.0	0.682
	2 years	90.1 ± 4.4	90.2 ± 4.9	89.0 ± 11.4	90.1 ± 5.0	0.983
KSS-FS	Preoperative	20.9 ± 5.6	19.7 ± 5.6	26.0 ± 8.2	21.9 ± 7.0	0.093
	1 year	87.7 ± 6.2	86.9 ± 6.9	86.0 ± 7.2	87.8 ± 5.8	0.872
	2 years	88.6 ± 7.0	87.2 ± 7.6	90.7 ± 11.4	87.4 ± 5.9	0.642
WOMAC	Preoperative	65.4 ± 7.6	65.3 ± 5.9	62.3 ± 3.5	62.8 ± 4.0	0.387
	1 year	26.2 ± 3.5	26.0 ± 4.3	24.7 ± 6.4	27.5 ± 3.5	0.473
	2 years	23.6 ± 3.9	23.4 ± 4.9	23.0 ± 7.9	24.4 ± 3.5	0.835
FJS	2 years	64.8 ± 7.9	63.5 ± 8.6	73.0 ± 7.0	69.8 ± 7.0	0.008
HFKS	2 years	35.3 ± 5.1	35.0 ± 4.9	38 ± 5.3	39.3 ± 3.4	0.005
Implant survival at 2 years	Any reason	100%	95.2% (95% CI, 88.8 – 98.0%)	100%	100%	0.343
	Aseptic loosening	100%	96.1% (95% CI, 90.0- 98.5%)	100%	100%	0.446

Presented as mean ± standard deviation (range). Abbreviations: preop, preoperative; postop, postoperative; n.r., not relevant; HKA, hip-knee-angle; KSS-KS, Knee Society Score - Knee Score; KSSFS, Knee Society Score - Function Score; WOMAC, Western Ontario and McMaster University Osteoarthritis Index; FJS, Forgotten Joint Score, HFKS, High Flexion Knee Score.
